# Determination of knowledge, attitude and practice of voluntary counseling testing on HIV among youths from Tepi Town, Ethiopia

**DOI:** 10.1016/j.pecinn.2022.100102

**Published:** 2022-11-15

**Authors:** Bizuwork Derebew, Misganaw Mola, Vikas Baliram Kalyankar, Nitin Devendra Padwal, Nitin Mahendra Chauhan, Atul Shivajirao Humbe, Sunil Tulshiram Hajare

**Affiliations:** a College of Natural and Computational Sciences, Mizan Tepi University, Mizan Tepi, Ethiopia; b Department of Zoology, Toshniwal Arts, Commerce and Science College, Sengaon, SRTMU, Nanded, India; c Department of Zoology, Shankarrao Patil Mahavidyalaya, Bhoom, Dist Osmanabad (MS), India; d Department of Biology, College of Natural and Computational Sciences, Dilla University, Dilla 419, SNNPR, Ethiopia; e Assist Prof & Head, Department of Zoology, SGRG Shinde College, Paranda, Dist. Osmanabad, India

**Keywords:** Attitude, Knowledge, Mizan Tepi, Practice, Voluntary counseling and testing

## Abstract

**Objective:**

Voluntary counseling and testing (VCT) is an integral component of HIV prevention and care strategies worldwide. VCT is considered as an effective strategy in risk reduction among sexually active youth. There is a high burden of HIV in Ethiopia despite a considerable scale up of comprehensive HIV interventions. Hence, the aim of this study was to assess the level of knowledge, attitude and practice of VCT uptake towards HIV among youth of rural part of Tepi town, Ethiopia.

**Method:**

A cross sectional study was carried out from May to August 2020. Total numbers of 127 youths in the age group of 15–24 years were recruited in a self-administered questionnaire with 100% response rate. Descriptive analysis was referred to describe the data. Binary logistic regression analysis was used to measure the effect of different variables by computing odds ratio. Multivariable analyses were utilized to determine the relative effect of explanatory variables.

**Results:**

The study showed that out of 127 total youth respondents, 111 (87.4%) of the respondents have good knowledge and 99 (78%) had a positive attitude towards VCT. Whereas, 86 (67.7%) of the respondents are aware of practicing VCT on HIV in the past.

**Conclusions:**

The findings revel that gender and father’s educational status were among the socio-demographic variables that showed statistically significant association with one or more variables affecting youths’ knowledge towards VCT uptake. As such, perceived risk of HIV infection, educational level, gender and marital status were statistically responsible for VCT uptake.

## Highlights

• The descriptive results showed that 89% of youths have knowledge towards VCT uptake
• 69.3% of youths have ever been tested for HIV voluntarily.
• Gender and fathers educational status were the most important determinant associated with knowledge of VCT uptake
• Perceived risk of HIV, educational level and marital status were the most important determinant associated with attitude of VCT uptake
• Sex and marital status were the most important determinant associated with practice of VCT uptake among youths

## 1 Introduction

Acquired immunodeficiency syndrome (AIDS) caused by the Human Immunodeficiency Virus (HIV) is a global burden of disease including Ethiopia [1]. Despite worldwide progress in antiviral therapy and implementation of awareness programmed approximately 2 million people become newly infected with HIV every year [2]. According to estimates, 722, 248 young Ethiopians contracted with HIV in 2017, with an increment of 3748 cases among young ones over the previous year [3]. 1, 037, 267 persons were reported to have HIV in Ethiopia, according to the Federal Ministry of Health [4].

The joint effort of World Health Organization (WHO) and the United Nations Program on HIV/AIDS (UNAIDS) in 1994, introduced a strategy called HIV voluntary counseling and testing (VCT) to prevent and control HIV/AIDS [5]. VCT provided an opportunity for both a primary and a secondary prevention by counseling before and after HIV testing [6].

Nowadays, the seriousness of the HIV/AIDS virus distribution has been acknowledged in Ethiopia and all the concerned bodies are making efforts to control the spread of the epidemic through different strategies. Among these strategies including VCT designed for prevention and control of the disease [7]. VCT is a globally recognized and successful method for treating HIV/AIDS affected individuals as well as preventing HIV/AIDS in infected society [8,9]. This service is cost-effective strategy for facilitating behavioral change and also an important entry point for care and support for HIV positive patients. Voluntary HIV/AIDS counseling and testing services provides people with an opportunity to learn and accept their HIV/AIDS status by building their self-confident with counseling and referral for on-going emotional support and medical care.

Beside the high levels of benefits of voluntary counseling and testing service, the utilization of VCT was low due to low numbers of VCT centers [10]. Helping to uninfected youth and save them from infection is a key strategy in the fight against AIDS and VCT has a major role to play in study area. It is observed that many youth assume they are infected when in fact they are not [11]. Many regional studies observed that when they learn they are not infected, not only is it a great relief for them, but it seems to help them to make changes in their sexual behavior to remain uninfected [12].

Most of the research findings in Ethiopia observed that utilization of voluntary counseling and testing service is low and it varies among different segments of the population [13]. According to CSA report [14], the rate of counseling and testing of HIV population was 37% and 38.2% in young people aged 15-24 years.

There are reports on the awareness and uptake of VCT service among higher secondary and university students in the study area [15,16]. However, the area lacks the information on the knowledge, attitude and practice of rural community towards VCT service. Therefore, present study was aimed to assess VCT service utilization and associated factors among the youths of Andinet village of Tepi town, Southwest Ethiopia.

## 2 Materials and methods

### 2.1 Study area and criteria

The proposed study was conducted from May to August, 2020 in Andinet village of Tepi town, Ethiopia. It is 611 km far from the capital city of Ethiopia i.e. Addis Ababa. The source of population includes youth of Andinet village between the ages of 15-24 years. All gender, race, and permanent residency were included in the proposed work.

### 2.2 Data collection

Data was collected by adopting self-administered structured questionnaires which comprised socio-demographic background of youth; there knowledge regarding VCT, attitude and practice towards VCT. The questionnaire was first prepared in English language and translated to the local language i.e. Amharic. Finally, the response was translated back to English language by independent translator to ensure for reliable information.The questionnaire was tested to retain consistency.

### 2.3 Sampling design

The samples were collected by using random sampling technique. The target population of this study was youths. After obtaining list of the youth from Tepi town government bureau office, selection of representative was done randomly. This applies to sampling without replacement i.e. once the youth was selected for the sample, it cannot appear in the sample again.

### 2.4 Sample size determination

Single population proportion formula was used to determine sample size. The prevalence rate (p=0.7) was taken from a previous study conducted in Bahir Dar University, Bahir Dar, Ethiopia [17] with the precision of 0.3%, d has been taken as half of p [18]. A sample size was calculated as below:

n=Z^2^(pq)/d2

Where, **Z**^**2**^**=**critical value for normal distribution at 95% confidence interval; **p=**proportion of prevalence; **d2=**marginal error. Hence, the size of our sample was calculated as follow:

n = (1.96)^2^*0.7*0.3/ (0.08)^2^

n=126.0525

By assuming n less than 0.05, the final sample size is calculated as follow:

n=127

### 2.5 Data scoring

Each question on VCT knowledge received a response of "Yes" for accurate answers or "No" for incorrect ones. All respondents' scores were added up, and the mean value was determined. The remaining participants were classified as unknowledgeable, while those who scored above or equal to the mean were termed knowing. The youth’s attitude toward the VCT exam was evaluated using an attitude indicator, which asked if the respondent agreed or disagreed with the test. After assigning a value of "1" or "0" for positive and negative replies, respectively, each of the attitudinal scores was added to create a score that served as a proxy variable. Respondents with scores above or equal to the proxy variable were thought to have positive attitudes, while those with scores below the proxy variable were seen to have negative attitudes. Using a single question with a "Yes" or "No" response, practice was evaluated. Those who said "Yes" were taken into account since they had previously used VCT services.

### 2.6 Analysis of data

Descriptive analyses were conducted to elucidate VCT for HIV. Level of associations between categorical variables was based on the chi-squared test. Three multiple logistic regression analyses were conducted separately to identify factors independently associated with knowledge of VCT, attitude and practice towards VCT. The model fitness for the logistic regression was tested with Wald test, Likelihood-Ratio test, Hosmer and Leme-show test and Omnibus test.

## 3 Results

### 3.1 Socio-demographic characteristics of the participants

Out of 127 participants, 66.9% were male and 63% of them had knowledge about VCT uptake. 33.8% had an illiterate father and most of them i.e. 30.7% had knowledge about VCT uptake (Table 1). The chi-square result showed significant association between status of father’s education and gender of the youths in VCT uptake at 5% level of significance and hence included in correlation analysis. Almost half of the respondent (50.4%) did not consider themselves at risk of HIV infection. About 18.1% were illiterate and 10.2% of them had positive attitude about VCT uptake. The adoption of VCT was positively impacted by marital status (49.6%), and the adoption of VCT was seen favorably by 39.4% of those surveyed. All of the factors such as gender, education and marital status had a significant impact on the uptake of VCT. Half of the male (51.2%) had practice of VCT uptake and 48.8% of the respondents were married out of them of which 32.3% respondents had practice for VCT uptake (Table 1).

**Table 1:** Chi-square analysis of knowledge, attitude and practice of VCT uptake on the selected explanatory variables among youths from Tepi town, Ethiopia.

Chi-square result of knowledge of VCT uptake						
Variable	Level	Knowledge of VCT uptake		Chi-square	DF	*P* value
		Yes: Count (%)	No: Count (%)			
Gender	Male	80 (63.0%)	5 (3.9%)	10.529	1	0.001
	Female	31 (24.4%)	11 (8.7%)			
Fathers education	Illiterate	39 (30.7%)	4 (3.1%)	12.751	2	0.002
	Primary	43 (33.9%)	1 (0.8%)			
	Secondary and above	29 (22.8%)	11 (8.7%)			

Chi-square result of attitude of VCT uptake						

Variable	Level	Attitude of VCT uptake		Chi-square	DF	*P* value
		Positive: Count (%)	Negative: Count (%)			

Perceived risk of HIV infection	Yes	55 (43.3%)	8 (6.3%)	6.358	1	0.012
	No	44 (34.6%)	20 (15.8%)			
Education level	Illiterate	13 (10.2%)	10 (7.9%)	7.728	2	0.021
	Primary	28 (22.0%)	7 (5.5%)			
	Secondary and above	58 (45.7%)	11 (8.7%)			
Marital status	Single	43 (33.9%)	7 (5.5%)	11.991	2	0.002
	Married	50 (39.4%)	13 (10.2%)			
	Other	6 (4.7%)	8 (6.3%)			

Chi-square result of practice of VCT uptake						

Variable	Level	Practice of VCT uptake		Chi-square	DF	*P* value
		Yes: Count (%)	No: Count (%)			

Gender	Male	65 (51.2%)	20 (15.7%)	9.010	1	0.003
	Female	21 (16.5%)	21 (16.5%)			
Marital status	Single	38 (29.9%)	11 (8.7%)	6.443	2	0.040
	Married	41 (32.3%)	21 (16.5%)			
	Others	7 (5.5%)	9 (7.1%)			

### 3.2 Multiple binary logistic regressions

Table 2 consist the parameter estimates value for multiple logistic regression model. To get the multiple logistic regressions, first we fitted the simple logistic regression model for each potential predictor with the knowledge of VCT uptake. Then we consider those predictor variables whose has the *P* value less than 25% to fit multiple logistic regression model as an important predictor variables and here we used the forward selection technique to determine the last significant variables to the model. Gender, educational status and marital status were considered for multivariate analysis All the variables were found to be significantly associated with VCT uptake (Table 2).

**Table 2:** Binary logistic regression on knowledge, attitude and practice of VCT uptake with selected independents variables for youths from Tepi town, Ethiopia.

Parameters estimated for binary logistic regression on knowledge of VCT uptake									
Name of variable	Level of the variables	B	S.E.	Wald	DF	Sig.	Exp(B)	95% CI for EXP(B)	
								Lower	Upper
Gender	Male Ref (female)	−1.906	0.625	9.296	1	0.002	0.149	0.044	0.506
Father Education status				9.711	2	0.008			
	Illiterate (1)	−1.435	0.677	4.484	1	0.034	0.238	0.063	0.899
	Primary (2) Ref (secondary and above)	−2.969	1.101	7.269	1	0.007	0.051	0.006	0.445
Constant		0.097	0.488	0.040	1	0.842	1.102	----	----

Parameters estimated for binary logistic regression on attitude of VCT uptake									

Name of variable	Level of the variables	B	S.E.	Wald	DF	Sig.	Exp(B)	95% CI for EXP(B)	
								Lower	Upper

Perceived risk	Yes Ref (no)	−1.294	0.516	6.298	1	0.012	0.274	0.100	0.753
Educational Status				8.836	2	0.012			
	Illiterate (1)	1.764	0.610	8.378	1	0.004	5.837	1.768	19.277
	Primary (2) Ref (secondary and above)	0.285	0.582	0.240	1	0.624	1.330	0.425	4.164
Marital status				10.379	2	0.006			
	Single (1)	−2.372	0.748	10.049	1	0.002	0.093	0.022	0.404
	Married (2) Ref (others)	−1.656	0.651	6.468	1	0.011	0.191	0.053	0.684
Constant		0.378	0.617	0.376	1	0.540	1.459	-------	----------

Parameters estimated for binary logistic regression on practice of VCT uptake									

Name of variable	Level of the variables	B	S.E.	Wald	DF	Sig.	Exp(B)	95% CI for EXP(B)	
								Lower	Upper

Gender	Male Ref (Female)	−1.087	0.420	6.690	1	0.010	0.337	0.148	0.768
Marital status				5.096	2	0.078			
	Single (1)	−1.317	0.643	4.190	1	0.041	0.268	0.076	0.946
	Married (2) Ref (others)	−1.318	0.638	4.264	1	0.039	0.268	0.077	0.935
Constant		−0.076	0.043	3.071	1	0.080	0.927	-------	-------

### 3.3 Model summary (Omnibus test, Hosmer-Lemeshow and model summery for the model)

Table 3 highlights the result for Omnibus test of model coefficient independent) variables to the model had not significant different from intercept only in the model (i.e. evaluating ability of predicting the variation in the model by independents variables) on the level of knowledge, attitude and practice of VCT uptake of to test that the selected potential predictor (youths. However, *P* value for Omnibus test less than 5% level of significance implying that, there is enough evidence to say that add those predictor variables in the model statistical significance different from intercept only model at moderate level. Thus, the influence of those add explanatory variable in predicting the outcomes probabilities were at moderate level.

**Table 3:** Model summary (Omnibus test, Hosmer-Lemeshow and model summary for the model) of selected variables for youths from Tepi town, Ethiopia.

Model summery for knowledge of VCT uptake				
Omnibus Test of Model Coefficients				
		Chi-Square	DF	Sig
1	Step	23.354	4	0.000
Hosmer and Lemeshow Test				
1	Step	12.503	8	0.130
Model Summery		−2 Log likelihood	Cox and Snell R Square	Nagelkerke R Square
1	Step	72.831	0.168	0.316

Model summery for attitude of VCT uptake				

Omnibus Test of Model Coefficients				
		Chi-Square	DF	Sig
1	Step	17.509	4	0.002
Hosmer and Lemeshow Test				
1	Step	5.178	7	0.638
Model Summery		−2 Log likelihood	Cox and Snell R Square	Nagelkerke R Square
1	Step	142.254	0.129	0.180

Model summery for practice of VCT uptake				

Omnibus Test of Model Coefficients				
		Chi-Square	DF	Sig
1	Step	27.038	6	0.000
Hosmer and Lemeshow Test				
1	Step	13.451	8	0.097
Model Summery		−2 Log likelihood	Cox and Snell R Square	Nagelkerke R Square
1	Step	106.948	0.192	0.294

Hosmer-Lemeshow test applied to assess overall goodness of fit the model to the data. The Hosmer-Lemeshow test is performed by dividing the partitioning the observation into g (usually to 10) equal sized groups according to their predicted probabilities and then computing a Pearson chi-square that compares the predicted to the observed frequencies. A non-significant Pearson chi-square test result (i.e. by comparing the *P* value to 5% level of significance) indicates that the model fit to the data.

Table 3 also depicts pseudo statistic (Log likelihood) in the evaluation of the model and the two different R^2^ measure the variations in the level of knowledge, attitude and practice of VCT uptake of the youths that can explain by selected explanatory variables. The results indicate that independent variables somewhere between 16.8%–31.6%, 19.2%–29.4% and 12.9%–18.0% of the variation in the level of knowledge, attitude and practice of VCT uptake of youths, respectively.

## 4 Discussion, innovation, conclusions

### 4.1 Discussion

The respondents who participated in this study were 15 to 24 years youth. This age group was selected because most of the youths particularly in this age accounts for over 50% of all HIV infections occurring worldwide. These youths are particularly vulnerable to HIV because of the strong influence of peer pressure and the development of their sexual and social identities, which often leads to experimentation. Youths should be counseled to delay their sexual debut and practice abstinence to avoid in contact to HIV [19]. In 2006, the population mostly affected by HIV in Ethiopia was those between the ages of 15–30 years. Of these, the majority of them were adolescents. There was a 5.6% prevalence rate of HIV infection among the group [20]. This indicates that age is an important demographic factor that should be given due attention in designing important prevention interventions for HIV infection.

A number of studies have been investigated the acceptability and practices of VCT among the school children in Ethiopia. None were specifically conducted to examine VCT uptake among rural youths in any other areas of Ethiopia, especially villages. From the findings of this study, the overall knowledge of respondents about VCT for HIV was high. The magnitude of knowledge towards VCT uptake in this study is similar with studies conducted in Jimma Town South Western Ethiopia (87.2%) [21]. High school students in Ethiopia discovered a similar story [22]. But this is relatively high compared to a study done in Bahirdar Town (80%) [17]. And this difference in magnitude with the study done in Bahirdar town could be their difference in socio demographic, basic environmental and behavioral characteristics of the respondents and which may be due to difference in sample size between two studies [17]. From the demographic and socioeconomic factors showed in Table 1 suggest that fathers educational level, gender, mothers educational level, religion, were not statistically associated, however, marital status, perceived risk and educational level are statistically significant with attitude of VCT uptake. Similar results were found from the study that conducted in Wolkite University, Ethiopia by Abdu et al. [23]. As the overall attitude score showed 78% of the youths have positive attitude towards VCT, lower results was reported compared with study from Debre Birhan, which was a 86% respondent had favorable attitude towards VCT [24].This may be due to difference in awareness between this two study areas. From the demographic and socioeconomic factors showed in Table 1 reflects that educational level, perceived risk, fathers educational level, mothers educational level, religion, were not showed significant association but marital status and gender are significantly associated with practice of VCT uptake. Majorities (67.7%) of respondents were practice of VCT Uptake; this finding is similar to Bahirdar study which reported 68%. The result of our study also indicates that female participants are the ones who are not knowledgeable, have a lower attitude, and practice towards VCT. This may be probably due to HIV rates are high among youth with HIV infected females being disproportionately affected, with a ratio to infected males in excess of 4:1 in some populations especially in the developing countries like Ethiopia [25].

Most of the respondents were aware for modes of HIV transmission. Un-precautionary sex with HIV positive individuals was the most common mode of HIV transmission cited by the respondents. This finding also emerged in the 2005 Ethiopian Demographic Health Survey among the youths [26]*.*Thus to prevent HIV transmission, it is important that young people practice safe sex through the much advocated ABC methods (abstinence, being faithful to one uninfected partner, and condom use) [26]*.* Knowledge of HIV transmission modes is particularly important for youths, since they are greatly threatened by the HIV pandemic and understanding how to prevent HIV transmission is the first step to avoid infection [27].

The highest prevalence of HIV is still found among the age group of between 15-24 years [28]. Attawell [29] reported that the highest HIV prevalence still occurs in the age group 15-30 years It is this same age group of women who are sexually active but never-married that has the highest rate of HIV infection and thus is expected to bring about a significant reduction in overall life expectancy [30]. A variety of factors are placing young people at the centre of HIV vulnerability and young people often begin their sexual lives at early ages [5]. HIV prevalence rates among youth reflect the realities of these risks. Altogether, HIV rates are high among youth [5]. The current study found that most of the youths are reluctant to go for testing and this place them and their partners at risk of being infected with HIV.

### 4.2 Innovation

In Ethiopia, HIV/AIDS is a serious health issue that affects people of all ages, but youths are particularly affected. Statistics show that the majority of those at risk are young people, with 60–80% of new HIV infections occurring in people between the ages of 15–24. Despite several VCT initiatives by the government and non-governmental organizations, the desire to undergo VCT is remains low in developing countries like Ethiopia. Teenagers' poor VCT uptake is a critical problem that requires attention especially at village level. There are reports on upper secondary and university students in the study area's knowledge of and use of VCT services. However, there is a dearth of data about the understanding, attitudes, and practices of rural communities regarding VCT services. These results showed that although Ethiopian adults are aware of VCT, there are obstacles preventing the programmed from being used to its full potential. The numbers highlight in the study indicates a threat that juvenile HIV prevalence poses, underscoring the need for research on HIV testing among this population. Due to the devastating effects of HIV/AIDS on young people in developing nations like Ethiopia, which frequently struggles with a lack of resources for the delivery of health services, education on VCT is one of the most effective ways to give young people the tools they need to stop the spread of HIV/AIDS. After learning that the youth’s limited use of VCT may be due to their lack of understanding of and access to necessary resources. The proper distribution of information and provision of services should be prioritized. As such, our study supports that VCT can play a major role in combating HIV as thus it is recommended to improve knowledge, attitudes and practices of youths towards HIV.

## 5 Conclusions

The descriptive results showed that 89% of youths have knowledge of VCT uptake, 78% of youths have attitude towards VCT uptake and 69.3% of youths have ever been tested for HIV voluntarily. In this study, chi-square test and binary logistic regression were used and it was noted that gender and fathers educational status were the most important determinant associated with knowledge of VCT uptake, perceived risk of HIV, educational level and marital status were the most important determinant associated with attitude of VCT uptake and gender and marital status are the most important determinant associated with practice of VCT uptake among youths (*P* < 0.05). This calls for actions on improvement youth awareness based on their gender classification and father educational status. Also, required knowledge on improvement youth way of thinking based on their perceived risk of HIV infection, marital status and educational status.

## Funding

No specific funding has been received for the proposed work. Hence, funding is not applicable

## Availability of data and materials

Not applicable.

## Authors contribution

Study design: NMC, STH. Conducted the study: BD, MM. Analyzed the data: NMC, STH, BD, MM. Wrote the paper: STH, VBK, NMC, NDP. Edited the manuscript: NMC, STH, ASH. All authors read and approved the manuscript for publication.

## Declaration of Competing Interest

All authors declare that they don’t have any conflict of interest.
